# An Extreme Case of Lethal Abdominal Compartment Syndrome in Pediatric Patient With Synovial Sarcoma

**DOI:** 10.7759/cureus.35212

**Published:** 2023-02-20

**Authors:** Andi Zhang, Christian Saliba, Justin Sobrino, Shin Miyata, Christopher Blewett

**Affiliations:** 1 Pediatric Surgery, Saint Louis University School of Medicine, Saint Louis, USA; 2 Pediatric Surgery, Sisters of St. Mary (SSM) Health Cardinal Glennon Children's Hospital, Saint Louis, USA

**Keywords:** decompressive laparotomy, intra-abdominal soft tissue tumor, synovial tumor, pediatric laparotomy, abdominal compartment syndrome

## Abstract

There is a relative paucity of literature on abdominal compartment syndrome (ACS) in children compared to adults and even less describing ACS in pediatric oncologic patients. We present this case of ACS in a 14-year-old patient to highlight the acuity of lethal consequences despite swift adequate management. Our patient is a 14-year-old male with a history of non-verbal autism and large synovial sarcoma of the left chest wall. He was admitted for scheduled inpatient chemotherapy and radiation. On day 3 of admission, the patient's clinical condition rapidly deteriorated, and a surgical abdomen was found on the exam. In the operating room (OR), massive gaseous distention of the stomach, small intestines, and colon were noted. A loop of small bowel was under such high pressure that the force of evisceration sheared the bowel from the associated mesentery. Due to the severity of the dilated bowel loops, we could not return the eviscerated bowel back inside the abdomen, which led us to leave the Abthera wound vac as sole coverage. The patient was transferred to the PICU, and medical treatment was aimed toward palliative care. The patient passed away three hours later. This case illustrates the acute and lethal nature of ACS in a less studied population, the pediatric oncologic patient. Prompt detection and treatment of ACS are essential for the management of critically ill pediatric patients, especially in those with space occupying tumors within the abdominal cavity. However, extreme presentations of ACS can have lethal consequences despite swift surgical intervention and adequate management.

## Introduction

Abdominal compartment syndrome (ACS) is the physiologic result of an elevation in intra-abdominal pressure (IAP) and associated organ dysfunction [1]. There is a relative paucity of literature on ACS in children compared to adults and even less describing ACS in pediatric oncologic patients. Studies have shown that adverse effects of elevated IAP occur at lower levels than previously thought, which may be difficult to measure, and can manifest suddenly as fulminant ACS, especially in patients with intra-abdominal malignancies [2]. In particular, pediatric patients with large abdominal malignancies do not show obvious early symptoms, and many are also unable to complain about the increasing physical discomfort. Additionally, pediatric oncologic patients present with tumors that are generally large at diagnosis and tend to develop rapidly, leading to a rapid elevation in IAP that can lead to ACS [3]. Thus, ACS in pediatric oncologic patients can often present suddenly and lead to rapid multiple-organ dysfunction, requiring emergent decompressive laparotomy. Untreated ACS is associated with mortality up to 90% if not recognized and treated early [1]. We present this case of ACS in a 14-year-old nonverbal patient with large synovial sarcoma to highlight the speed of lethal consequences despite swift adequate management.

## Case presentation

**Figure 1:**
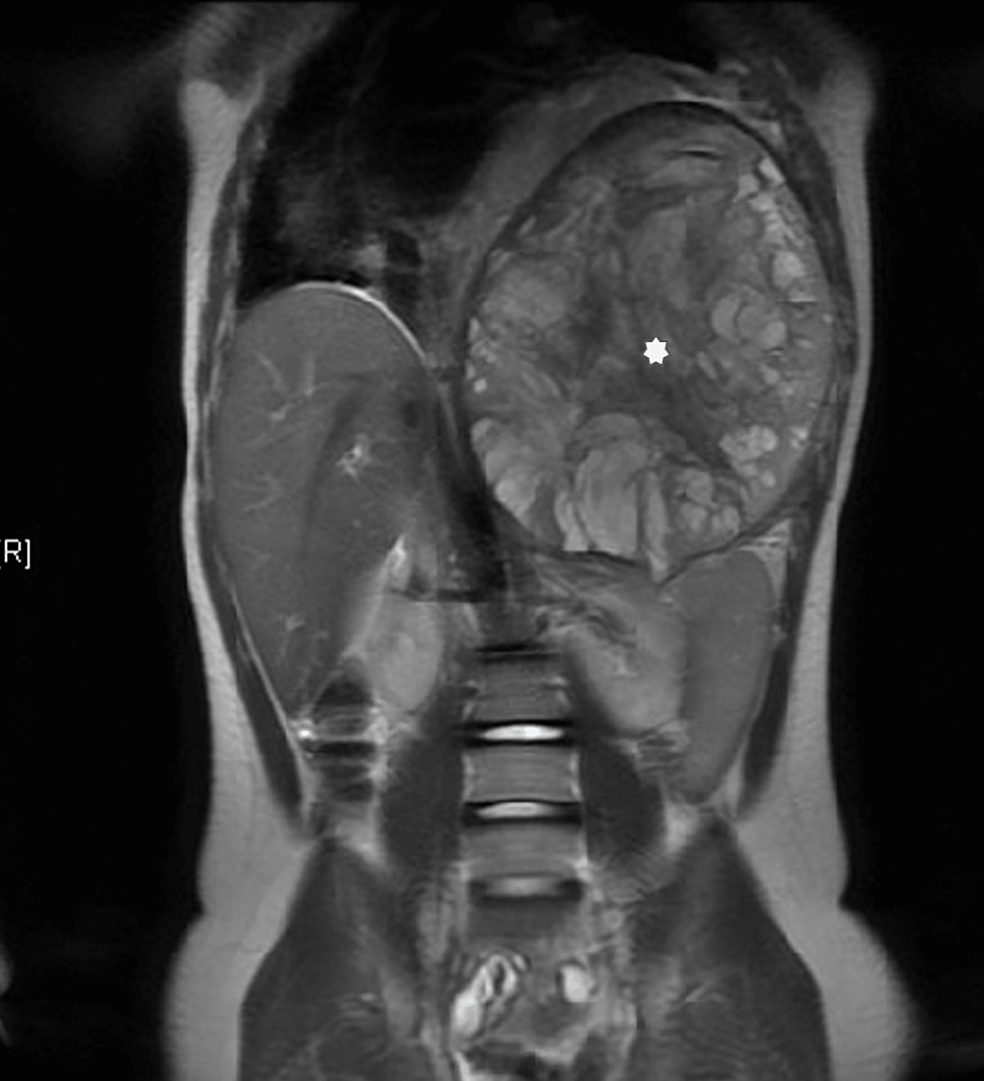
Large T2 hyperintense mass involving the left lower thorax, diaphragm, and chest wall extending into the left upper abdomen. It was taken one month prior to the current admission (Cor T2 SSFSE). Mass is labeled by a white star.

Our patient is a 14-year-old male with a history of non-verbal autism, obsessive-compulsive disorder, and large synovial sarcoma of the left chest wall. He was admitted for scheduled inpatient chemotherapy. Figure 1 shows the large synovial sarcoma of the left chest wall (15.6 x 18.3 x 22.4 cm anteroposterior [AP] x transverse [TV] x craniocaudal [CC] planes) on MRI imaging one month before this hospital admission. His week 7 chemotherapy regimen consisted of ifosfamide with mesna for three days and doxorubicin with dexrazoxane for two days, followed by pegfilgrastim for one day. Radiation was planned to start on day 4, at least 24 hours post-doxorubicin completion. However, due to agitation on day 1 of admission, chemotherapy was postponed until day 2. 

**Figure 2:**
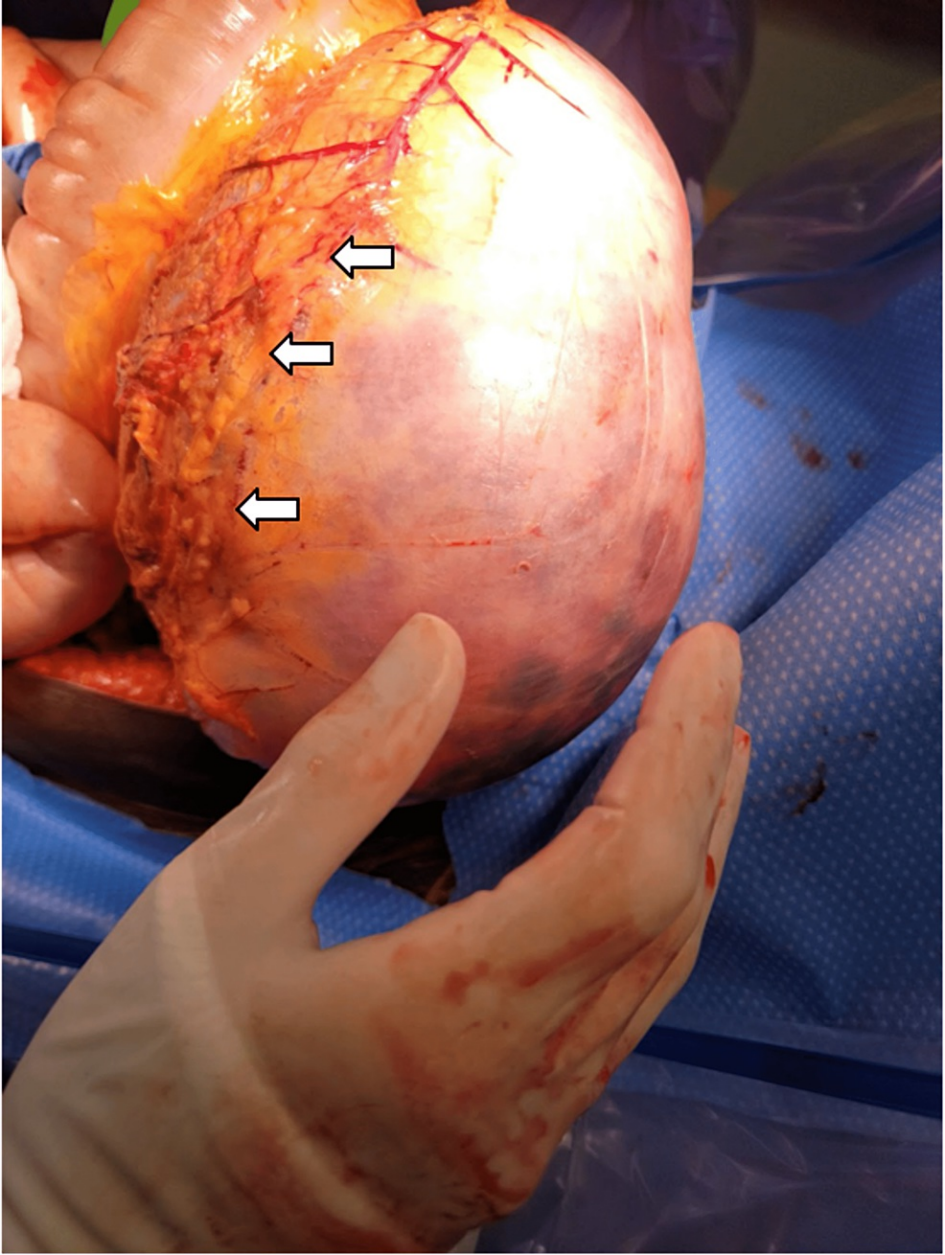
White arrows showing diffuse air embolism within the left gastroepiploic artery.

**Figure 3:**
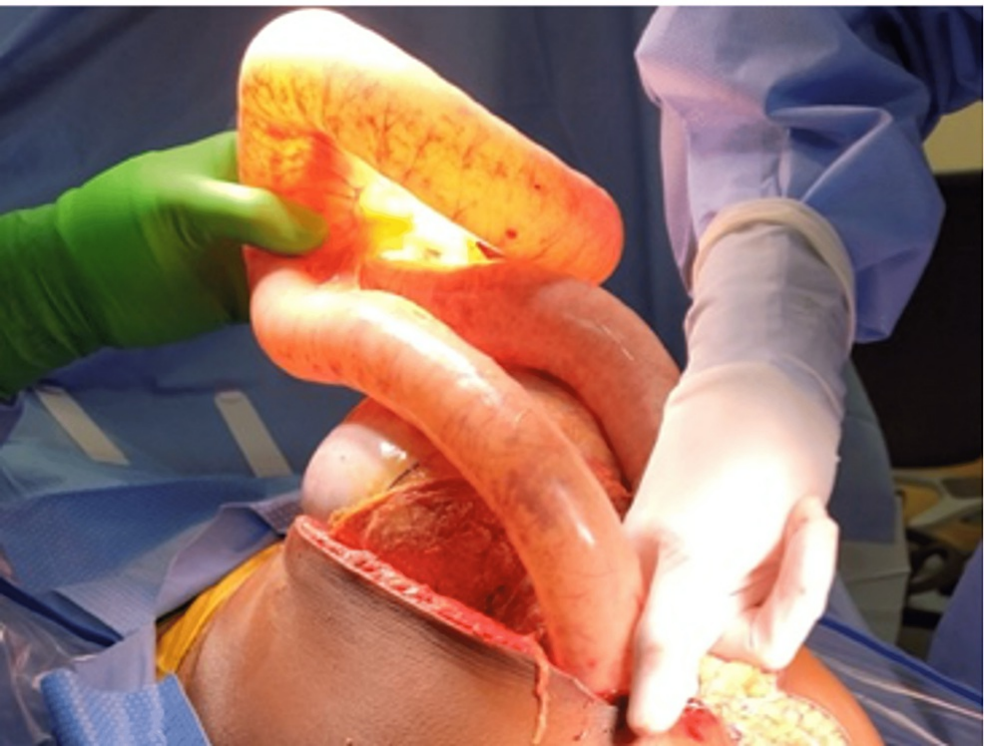
Massive gaseous distention of the stomach and small intestine.

**Figure 4:**
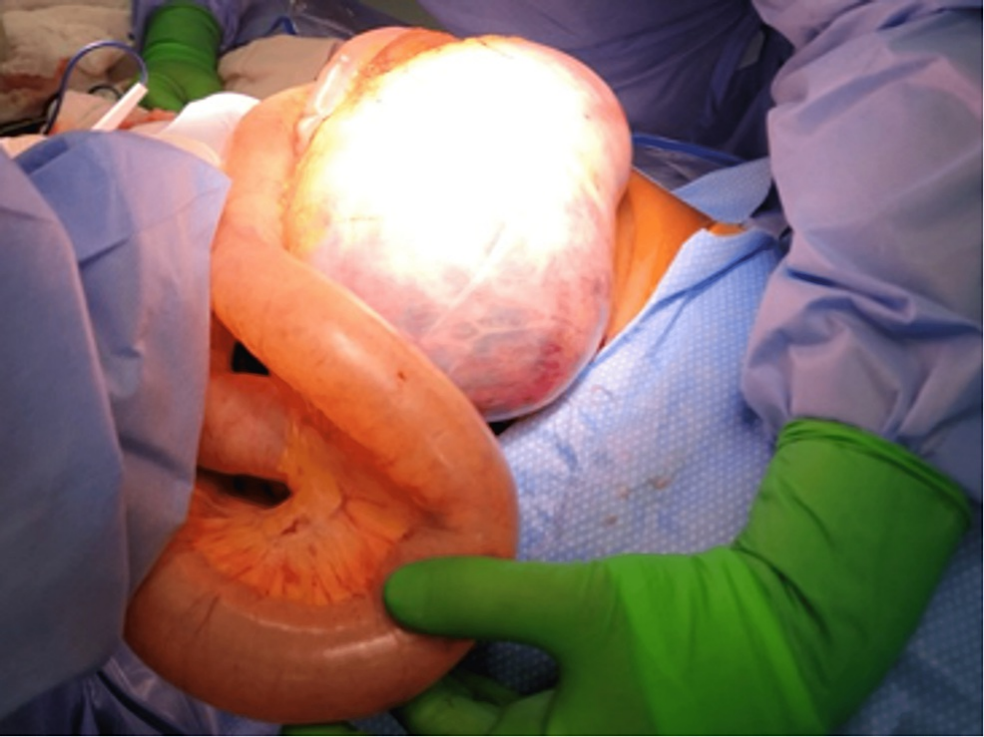
Massive gaseous distension of stomach (center of image) and small intestines from another angle.

**Figure 5:**
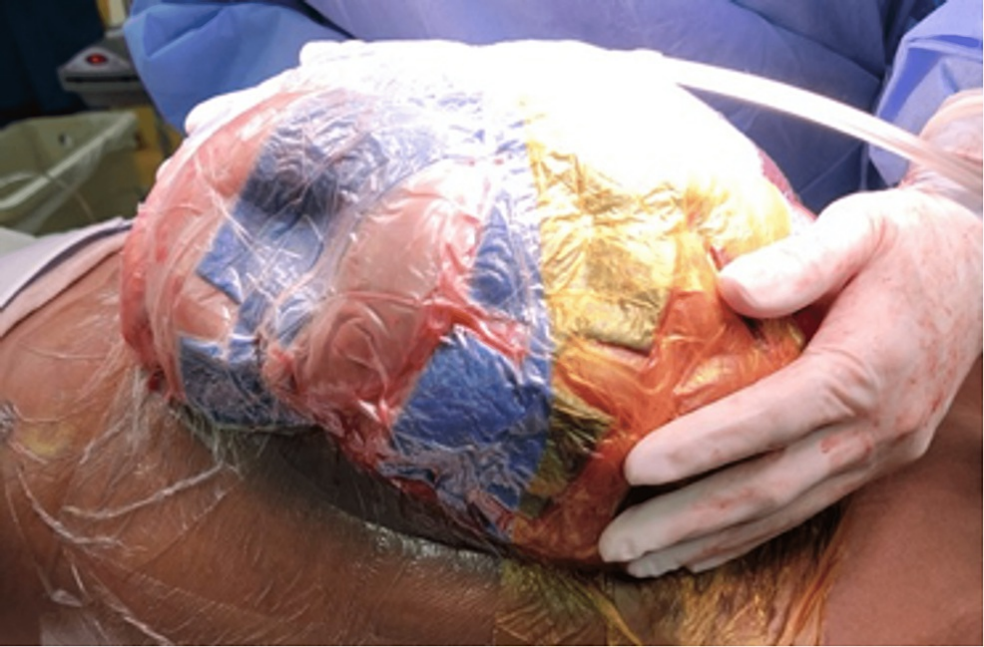
AbThera Open Abdomen Dressing.

During day 1 of admission, the patient remained hemodynamically stable with periods of agitation requiring PRN Thorazine along with security. On day 2, chemotherapy was started, along with continued agitation requiring several scheduled psychiatric medications. On day 3 of his hospital admission, his condition rapidly deteriorated in the morning. Overnight, the patient de-accessed himself and had agitation through the night, and after that, he was unable to get himself re-accessed. Around 4:30 AM, a distended abdomen was noted, and a kidney, ureter, and bladder (KUB) X-ray was ordered. Later in the morning, he was noted to be markedly distended and grunting, with poorly palpable pulses. He was also much less responsive than before. A rapid response was called due to concern for the clinical decline of tachycardia, hypoxemia, low blood pressure, and marked abdominal distention, evident on our physical exam of a guarded surgical abdomen. The patient's pre-operation labs included a worsening capillary blood gas pH 6.96 (N: 7.35-7.45 pH), pO2 150 (N: 80-100 mmHg), pCO_^2^_ 40 (N: 35-45 mmHg), HCO_3_ 9 (N: 20-30 mmol/L) lactic acid >16.9 (N: <2 mmol/L), complete blood count WBC 41.8 (N: 4.5-14.5 x 109/L) Hgb 7.2 (N: 13-16 g/dL) Hct 24.4 (N: 37-49%), phosphorus 10.1 (N: 3-6 mg/dL), amylase 115 (N: 5-65 U/L), lipase 52 (N: 8-78 U/L), lactic acid blood 2.5 (N: <2 mmol/L), type and screen panel: ABO (Rh) B(+) antibody negative. The patient was promptly transported to the OR for exploratory laparotomy. In the OR, massive gaseous distention of the stomach, small intestine, and colon under extreme tension were noted (Figures 2-4). A loop of the small bowel was under such high pressure that the force of evisceration sheared the bowel from associated mesentery (Figure 3). Air emboli were visualized in the gastroepiploic and mesenteric vessels (Figure 2). No clear bowel obstruction or tumor hemorrhage was identified. The patient notably had diffuse GI tract advanced ischemia, and this condition was deemed nonsurvivable. Due to the severity of the dilated bowel loops, we could not return the eviscerated bowel inside the abdomen, leading us to leave the AbThera open wound dressing as sole coverage (Figure 5). The patient was transferred to the PICU, and the patient passed away three hours later after a discussion with the family, and medical intervention was aimed towards palliative care.

## Discussion

ACS is defined as IAP greater than 20 mmHg and is associated with new end-organ dysfunction [3]. However, children have a lower baseline of IAP compared to adults, so ACS may occur when IAP reaches just 10 mm Hg [4]. In addition to the lower threshold for IAP, children who are critically ill and are limited in their ability to communicate physical symptoms further complicate the diagnosis of ACS [5]. While many attempts have been made to improve and/or simplify IAP measurement, research has shown that none of these novel techniques is truly superior to the standard, transvesical route. Additionally, biomarkers and imaging have also been tested, which were also not ready to replace traditional IAP measurement for the diagnosis of IAH or ACS [6]. Current treatment guidelines remain largely unchanged, with nonoperative management options as the preferred strategy for most patients. When surgery is indicated, it should be tailored to the cause of IAH. It mainly involves three therapeutic goals: evacuation of the causal intra-abdominal content, treatment of intra-abdominal injuries, and administration of fluids to maintain adequate perfusion of tissue and/or organs [4]. Decompressive laparotomy is generally followed by temporary abdominal wall closure 5-7 days after surgery and then with reconstruction performed 6-12 months after the last operation [7]. In children with large intra-abdominal tumors who are already quite ill, they do not present with obvious physical exam findings, and their organ dysfunction is often incorrectly attributed to the progression of their primary illness. Additionally, pediatric oncologic patients present with tumors that are generally large at diagnosis and tend to develop quickly, leading to rapid elevations in IAP that can easily progress to ACS [3]. There is a paucity of literature on ACS in children with abdominal tumors relative to adults, and even less in the setting of soft tissue malignancies such as synovial sarcoma. Due to the lack of literature beyond case studies, there is also a lack of consensus regarding the definition and general incidence of ACS in the pediatric population, further complicating its identification and management [8]. Other than a marked distended abdomen, other physical findings such as oliguria and increased ventilatory requirement can be challenging to differentiate from baseline in a critically ill patient with a rapidly growing intra-abdominal tumor. In patients suspected to have elevated IAP, routine monitoring is advocated. However, these patients often have common risk factors for ACS, such as trauma, massive hemorrhage, or protracted volume resuscitation situations [2]. Intra-abdominal malignancies are a less common cause of ACS, and thus routine monitoring of IAP is not routinely done and is highly dependent on physician judgment. Our case is atypical in the speed at which ACS presented. On the third day of hospital admission, the patient acutely decompensated and presented with a surgical abdomen which was not evident on the physical exam hours prior. Even though a decompressive laparotomy was done swiftly, the extreme build-up of gas within the bowels causing diffuse air embolism (Figures 2-4) prevented any surgical intervention from restoring organ perfusion.

## Conclusions

This case illustrates the acute nature of ACS and its presentation beyond trauma, massive hemorrhage, or protracted volume resuscitation situations in which ACS most often presents. Adverse effects of elevated IAP can go unnoticed for many days prior to the development of fulminant ACS. Prompt detection and treatment of ACS are essential for the management of critically ill pediatric patients, especially those with space-occupying tumors within the abdominal cavity. However, extreme presentations of ACS can prevent surgical intervention from achieving adequate hemodynamic restoration.
